# Progress in Obstetrics

**Published:** 1902-03-15

**Authors:** 


### PROGRESS IN OBSTETRICS.

(*Continued from page* 390.)

**Uterine Retraction.**—Dr. Berry Hart,[4] in a paper on this subject, deals with uterine retraction with special reference to the mechanism and management of the third stage of labour. He holds the view that the placenta is separated not during the "pain," but after it, and therefore advocates an expectant treatment during separation in the third stage of labour. By uterine retraction he understands the thickening which the uterine muscle retains after the contraction or "pain" is over. There is less retraction and thickening of the uterine wall over the placental site (before the separation of the placenta) than there is in other parts of the uterine wall. Gerlach[5] also thinks that normally the placental site does not, during labour, thicken itself by contraction and retraction to the same extent as do other portions of the uterine wall; and when after the birth of the child the placenta remains adherent, the portion of the uterine wall to which it is attached also remains thin. Should this placental site still fail to contract after the placenta has been removed, it may be pressed into the uterine cavity by the contracting muscle surrounding it, when it may be recognised as a rounded prominence with a corresponding depression on the outer side of the uterine wall. This condition of failure to contract and retract of that part of the uterine wall to which the placenta is attached is what is meant by paralysis of the placental site. It is doubtless a cause of inversion of the uterus, and from the non-closure of the vessels opening on the placental site it permits of profuse post-partum hæmorrhage. Horrocks[6] writing on this subject says that (1) retraction is the condition of a muscle which, having been shortened by contraction, the active contraction has given place to simple relaxation without extension; (2) in a state of retraction there is a slight and constant contraction, called tone; (3) retraction of the uterus in labour affects only the expulsive fibres of the fundus and upper two-thirds of the body of the uterus. It keeps the sinuses and broken blood-vessels of the placental site closed after the separation of the placenta, and is therefore of the greatest importance.

**Ovarian Pregnancy.**—Until within the last few years not only were cases of ovarian pregnancy regarded with scepticism, but doubts were thrown on the possibility of ovarian gestation in any case. Recently, however, a number of cases have been recorded which seem to show beyond doubt that such a condition as ovarian pregnancy is possible. Hastings Gilford[7] has collected and analysed the records of sixteen cases of undoubted ovarian pregnancy, and twelve probable cases; and he discusses the question as to how such an event as conception within a Graafian follicle can take place. One explanation is that the sperm cell enters through the aperture produced by the bursting of a follicle and impregnates an ovum which has not yet escaped. The opening then heals over and the pregnancy goes on in the sealed sac. The other hypothesis is given by Schroeder, who believes that the spermatozoon may possibly penetrate through the coat of the follicle at its most attenuated part. Of these explanations the former is, he thinks, by far the more probable, though it does not necessarily follow that the rent in the follicle closes. Impregnation may in some cases take place in a follicle which remains open.

[4] Scottish Med. and Surg. Jour., 1901, vol. viii. [5] Med. Chron., Aug. 1901. [6] The Jour. of Obstet. and Gynæcology of the British Empire, Jan. 1902. [7] Brit. Med. Jour., Oct. 5. 1901.

